# Novel Rhizosphere Soil Alleles for the Enzyme 1-Aminocyclopropane-1-Carboxylate Deaminase Queried for Function with an *In Vivo* Competition Assay

**DOI:** 10.1128/AEM.03074-15

**Published:** 2016-02-05

**Authors:** Zhao Jin, Sara C. Di Rienzi, Anders Janzon, Jeff J. Werner, Largus T. Angenent, Jeffrey L. Dangl, Douglas M. Fowler, Ruth E. Ley

**Affiliations:** a Department of Molecular Biology and Genetics, Cornell University, Ithaca, New York, USA; b Department of Microbiology, Cornell University, Ithaca, New York, USA; c Department of Biological and Environmental Engineering, Cornell University, Ithaca, New York, USA; d Howard Hughes Medical Institute, Chevy Chase, Maryland, USA; e Department of Biology, University of North Carolina, Chapel Hill, North Carolina, USA; f Department of Microbiology and Immunology, University of North Carolina, Chapel Hill, North Carolina, USA; g Carolina Center for Genome Sciences, University of North Carolina, Chapel Hill, North Carolina, USA; h Department of Genome Sciences, University of Washington, Seattle, Washington, USA; i Max Planck Institute for Developmental Biology, Tübingen, Germany; Stanford University

## Abstract

Metagenomes derived from environmental microbiota encode a vast diversity of protein homologs. How this diversity impacts protein function can be explored through selection assays aimed to optimize function. While artificially generated gene sequence pools are typically used in selection assays, their usage may be limited because of technical or ethical reasons. Here, we investigate an alternative strategy, the use of soil microbial DNA as a starting point. We demonstrate this approach by optimizing the function of a widely occurring soil bacterial enzyme, 1-aminocyclopropane-1-carboxylate (ACC) deaminase. We identified a specific ACC deaminase domain region (ACCD-DR) that, when PCR amplified from the soil, produced a variant pool that we could swap into functional plasmids carrying ACC deaminase-encoding genes. Functional clones of ACC deaminase were selected for in a competition assay based on their capacity to provide nitrogen to Escherichia coli *in vitro*. The most successful ACCD-DR variants were identified after multiple rounds of selection by sequence analysis. We observed that previously identified essential active-site residues were fixed in the original unselected library and that additional residues went to fixation after selection. We identified a divergent essential residue whose presence hints at the possible use of alternative substrates and a cluster of neutral residues that did not influence ACCD performance. Using an artificial ACCD-DR variant library generated by DNA oligomer synthesis, we validated the same fixation patterns. Our study demonstrates that soil metagenomes are useful starting pools of protein-coding-gene diversity that can be utilized for protein optimization and functional characterization when synthetic libraries are not appropriate.

## INTRODUCTION

Competition assays allow for a massively parallel assessment of the relative fitness of variants in a functional context (1). Variant pools can be generated synthetically or harvested from the environment. Recently, deep mutational scanning was developed as a method to elucidate the sequence-function relationships and optimal catalytic sequences of proteins (2, 3). Using a doped DNA oligomer library as a starting point for selection assays, followed by next-generation sequencing, Fowler et al. (2, 3) mapped the mutational preferences of hundreds of thousands of protein variants for an important human protein domain and thereby assessed the fitness effects of nearly all the possible point mutations in the protein domain. This method is able to assay truly novel mutations and combinations of mutations affecting enzyme function, thereby helping to generate optimized engineered proteins for biomedical or other use.

The use of artificially produced proteins may be constrained for ethical and social reasons, particularly in the agricultural arena. An alternative to selection based on artificially produced proteins is to select for naturally occurring protein diversity. Naturally occurring environmental microbial communities, which contain a high diversity of protein variants (4–6), might provide an attractive source of gene variants for use in structure-function studies or enzyme optimization (7–9). Soil metagenomes, in particular, comprise a rich and mostly uncharacterized reservoir of protein-coding gene diversity encoded by a vast diversity of microorganisms (9). Metagenomes have been mined for natural product discoveries, including novel proteins that function as antibiotics (6) and cellulose-degrading enzymes (10). Such proteins are typically encoded by many homologs, which are likely to vary in their functional attributes (11). Here, we asked a simple question: can a soil metagenome be used as a starting point for a protein selection assay?

To address this question, we designed a study in which we used a growth-based competition assay to investigate the soil enzyme 1-aminocyclopropane-1-carboxylate deaminase (ACCD), which catalyzes the degradation of 1-aminocyclopropane-1-carboxylate (ACC) to α-ketobutyrate and ammonia (12). ACC is a key intermediate in the production of the plant growth hormone ethylene (13). The agricultural control of ethylene levels has proven crucial for minimizing the destructive effects of environmental stresses, including salt (14), water (15), and heavy metals (16), and in promoting root elongation (17, 18). Due to its critical role in promoting plant growth, ACCD has been identified as a key target for bioprospection (17).

ACCD is expressed by bacteria associated with soil surrounding plant roots, or the rhizosphere (19). Although the ACCD gene is carried by members of divergent taxa, predominantly those belonging to the phyla Proteobacteria, Firmicutes, and Actinobacteria, regions of the protein are well conserved (20). ACCD is composed of a pyridoxal 5′-phosphate (PLP)-dependent (21) active site surrounded by large and small domains. The active site is situated in a well-conserved region, the ACCD domain region (ACCD-DR) (21–24). ACCD DNA sequences, however, have been recovered in bacteria and plants lacking ACC deaminase activity (17). Therefore, functional assays must be applied to confirm ACC deaminase function.

ACCD is a unique enzyme, in that it allows for bacterial growth when ACC is the only source of nitrogen (25). Therefore, we tested whether soil microbiome-derived ACCD gene variants in a bacterial strain competition assay conducted with ACC as the sole nitrogen source would emerge and be indicative of enhanced ACCD activity in this growth context. We observed dominant variants in the competition assay, which confirms that a soil microbiome can be used as a starting point in a selection assay. Based on the selection results, we predict residues to be functional, divergent, or neutral within the ACCD-DR, and these results are largely in agreement with previous structure-function analyses. Of particular note, we uncovered diversification at a previously identified essential residue, hinting at alternative substrates or structural conformations that might be accommodated by ACCD. By using a wide pool of variants versus single mutations, we were also able to capture combinations of residues that appear to have undergone collective selection and therefore may function cooperatively. Finally, we validated the use of a soil microbiome library for protein optimization, as we obtained similar results from competition among synthetic ACCD-DR variants generated by doped DNA oligomer synthesis. Altogether, this work highlights the structural and evolutionary knowledge that can be gleaned by assessing the sequence variants present in a natural sample.

## MATERIALS AND METHODS

### Initial assessment of ACCD-DR diversity in rhizosphere soil.

**FIG 1:**
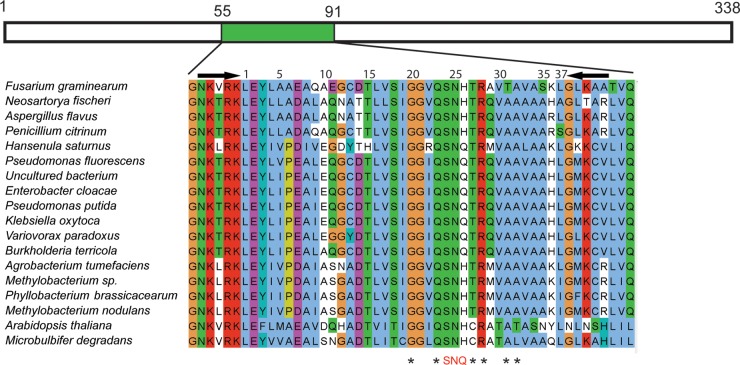
Alignment of ACCD-DR from various organisms. Shown is a Clustal X-colored alignment of characterized ACC deaminase proteins in 18 organisms from bacteria, fungi, and plants (sequence identifiers are provided in the supplemental material). The full-length ACC deaminase is shown above the alignment, and the ACCD-DR is marked in green. The amino acid numbering is based on the full-length Pseudomonas putida ACC deaminase. The arrows show the locations of the primers used in this study to amplify the ACCD-DR. The 37 residues of ACCD-DR are numbered inside the primer-amplified region. SNQ, structurally identified active-site residues binding cofactor and sulfate; asterisks represent other known conserved residues in ACCD-DR identified in the reports of Karthikeyan et al. (23) and Yao et al. (21).

Rhizosphere soil samples from four maize inbred lines (Oh43, MS71, M37W, and NC358) were collected from a field in Missouri, USA, in 2010, and DNA was extracted as previously described (26). PCRs used the custom primers a2F (5′-GSAACAAGACGCGCAAG-3′) and a2R (5′-CACSAGCACGCACTTCATG-3′), which amplify a 37-amino-acid region from the full-length ACCD gene (Fig. 1). These degenerate primers were designed using an alignment of bacterial ACCD genes obtained from public databases. PCR mixtures (25 μl) consisted of 10 ng of rhizosphere DNA, a 0.16 μM concentration of the primers a2F and a2R, 1× GoTaq buffer (Promega Corporation, Madison, WI), 0.001 U GoTaq, 2 mM Mg^2+^, and 0.2 mM deoxynucleoside triphosphates (dNTPs). Thermal cycling consisted of an initial denaturation at 95°C for 2 min, 30 cycles of denaturation at 95°C for 1 min, annealing at 52.9°C for 50 s, and elongation at 72°C for 1 min, followed by a final extension at 72°C for 7 min. The amplicons were purified with the QIAquick PCR purification kit (Qiagen, Valencia, CA) and pooled for sequencing. The addition of an Illumina linker and adaptor sequences and sequencing on the Illumina genome analyzer IIx (Illumina, Inc., San Diego, CA) were conducted by the Cornell University Biotechnology Resource Center.

The resulting Illumina sequence reads were processed using in-house perl scripts. Paired-end sequences were joined based on alignment of the 23-bp overlapping region, with no internal gaps allowed. The reads were filtered by trimming low-quality bases (Q20 cutoff) from single-direction reads and discarding reads that were trimmed >6 bases. Up to three tailing bases (for each direction) disagreeing with the complementary sequence were allowed to be trimmed. We confirmed that the trimmed tailing bases had comparatively lower quality scores and likely lower sequencing errors than the complementary sequence. The ACCD-DR DNA sequences were translated to their corresponding amino acid sequences. Amino acid sequences were then clustered by absolute identity using UCLUST (27) to tabulate the protein-level diversity available in the soil sample pool of variants.

### Generation of ACCD plasmids lacking the ACCD-DR.

To assay the ACCD-DR variants amplified from the rhizosphere, we first generated plasmids bearing the ACCD gene but lacking the ACCD domain region. A plasmid, p4U2, containing the Pseudomonas cloacae ACCD and its flanking region (28), was obtained as a generous gift from Bernard Glick, University of Waterloo, Ontario, Canada. We deleted the ACCD-DR from p4U2 using the primer acdSdelF (5′-AATAGCGGCCTGGCCTTCGGCGCAGGAAAACTGGGTGAACTACT-3′) and the QuikChange Lightning site-directed mutagenesis kit (Agilent Technologies, Santa Clara, CA). This PCR mixture was 51 μl containing 1× QuikChange reaction buffer, 50 ng of p4U2 plasmid DNA, 0.2 μM acdSdelF primer, 1 μl QuikChange dNTP mixture, 1.5 μl of QuikSolution reagent, and 1 μl of QuikChange Lightning enzyme. Thermal cycling consisted of an initial denaturation at 95°C for 2 min and 18 cycles of denaturation at 95°C for 20 s, annealing at 60°C for 10 s, and elongation at 68°C for 5 min, followed by a final extension at 68°C for 5 min. The plasmid without the P. cloacae ACCD-DR is referred to as p4U2ΔACCD-DR.

### Construction of Escherichia coli ACCD-DR variant libraries.

To amplify and sequence the ACCD-DR by 454 pyrosequencing, we added the 454 adaptors to the above-mentioned a2F/a2R primers and generated the following primer set: acdSinserF (5′-AATAGCGGCCTGGCCTTCGGCG**GSAACAAGACGCGCAAG**-3′) and acdSinserR (5′-CGGAGTAGTTCACCCAGTTTTCCTG**CACSAGCACGCACTTCATG**-3′), where the bold regions indicate the primer sequences used to amplify the ACCD-DR, and the nonbold type sequences are the 454 adaptors. For the library construction, we used DNA extracted from a soil sample obtained from B73 maize grown in Lansing, NY (26).

**FIG 2:**
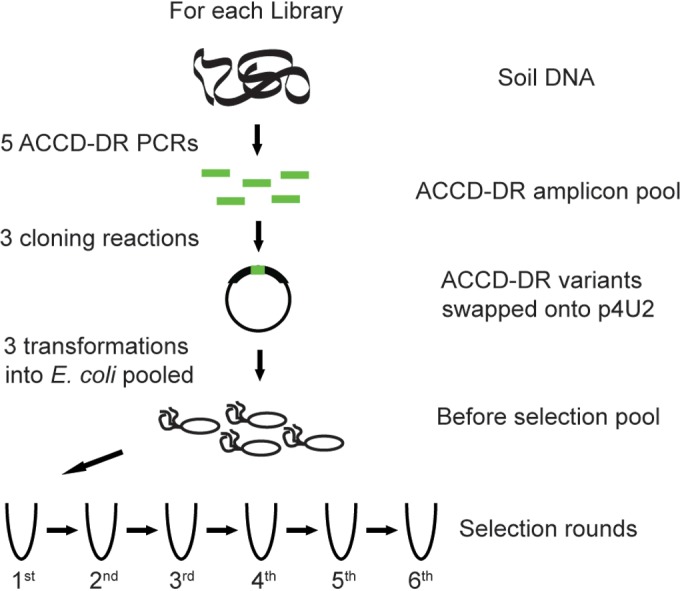
Construction of E. coli ACCD-DR variant libraries and growth-based selection assay. For each ACCD-DR variant library, the ACCD-DR was amplified from soil DNA in five separate PCRs. This combined amplicon pool was cloned in triplicate into p4U2 to replace the ACCD-DR on the plasmid. From each cloning reaction, the clones were transformed in triplicate into E. coli and subsequently pooled during a liquid culture grow-out. This pool is used as the “before selection” pool. These E. coli strains were passaged through the growth-based selection assay six times. Note that the same soil DNA was used for each library (A, B, or C). See Materials and Methods for further details on the library construction and selection assay.

To capture the diversity of ACCD genes from the rhizosphere, we performed 15 separate PCRs and pooled the results in groups of five, for a total of three pools. These pools are referred to here as libraries A, B, and C (see Fig. 2 for an overview of the library construction and competition assay). For each library, three separate cloning reactions were used to insert the amplicons into p4U2ΔACCD-DR using the QuikChange Lightning site-directed mutagenesis kit, similar to what is described above. The PCR conditions were as described above, and for each library, amplicons from the five replicate PCRs were combined and purified using the QIAquick PCR purification kit (Qiagen, Valencia, CA).

Each library plasmid pool was then transformed into E. coli XL10-Gold chemical ultracompetent cells (Agilent Technologies, Santa Clara, CA), according to the manufacturer's protocol, in triplicate. The three transformations were then combined (total volume, 1.65 ml), lysogeny broth (LB) supplemented with 50 mg/ml ampicillin was added to a final volume of 5 ml, and cells were grown at 37°C overnight in a shaking incubator. The overnight culture was spun down and washed twice in 0.1 M Tris-HCl buffer (pH 7.5) to prevent carryover nitrogen. The washed cell pellets were resuspended in 1.65 ml of Dworkin and Foster (DF) minimal medium (29) minus (NH_4_)_2_SO_4_ and supplemented with 0.2% dextrose, 50 mM MgSO_4_, 1 mM CaCl_2_, 50 mg/ml ampicillin, and 10 μg/ml thiamine. The washed overnight cultures (300 μl) were frozen as “before selection” ACCD-DR pools.

### Growth-based competition assay.

Resuspended cell pellets (300 μl, normalized by the optical density at 600 nm [OD_600_] values of the previous round of cultures) were added to 30 ml of supplemented DF minimal medium minus (NH_4_)_2_SO_4_ in three replicates, and ACC was added to the medium as the sole nitrogen source at a final concentration of 16 mM. Here, this growth medium is called the DF/ACC medium. These E. coli cells were grown at 30°C for 5 days in the first round of selection. At the end of the first round of selection, the cultures were harvested, spun down, washed, and resuspended. The washed and resuspended cells (300 μl, normalized by the OD_600_ values of the previous round of cultures, as described above) were again transferred into 30 ml of fresh DF/ACC medium to start the second round of selection. The second round of selection consisted of 3 days of growth at 30°C. Cells were passaged into fresh DF/ACC medium to start the third round of selection in a similar manner. A total of six rounds of selection were conducted on each ACCD-DR variant library. Importantly, 300-μl volumes of cultures were collected as ACCD-DR variant pool samples after each round of selection for sequencing.

E. coli cells containing different rhizosphere ACCD-DR variants had heterogeneous growth rates within each variant library and between libraries; therefore, we did not synchronize the E. coli cells to the same growth stage but rather ensured that we provided the same amount of E. coli cells for each round of selection across all three variant libraries based on normalized OD_600_ values, and we gave each library of variants the same growth time. Library B was assayed only in duplicate because the time zero culture was lost for one of the pools.

To test for cheaters (i.e., E. coli cells growing without a functional ACC deaminase on the nitrogen produced by E. coli cells with a functional ACC deaminase), we performed the competition assay in a similar manner, except that three rounds of selection were conducted.

### DNA oligomer synthesis for the artificial ACCD-DR variant pool.

A DNA oligonucleotide variant pool was synthesized, as described previously (2) (Gene Link, Hawthorne, NY). The DNA sequence of the winning LA variant (CTCGAATACCTGATCCCCGAGGCGCTGGCGCAGGGCTGCGACACGCTGGTGTCGATCGGCGGCATCCAGTCGAACCAGACACGCCAGGTTGCGGCCGTGGCTGCCCACCTGGG, which encoded LEYLIPEALAQGCDTLVSIGGIQSNQTRQVAAVAAHL), was chosen as the wild-type backbone of the oligonucleotide and doped at each base with 2.1% non-wild-type nucleotides. The growth-based competition for the artificial variant pool was conducted as described above for the rhizosphere-derived ACCD-DR library.

### Sequencing of ACCD-DR variants from the competition assays.

Plasmid DNA was extracted from the ACCD-DR variant pools collected before and after each round of selection using the QIAprep spin miniprep kit (Qiagen, Valencia, CA). The ACCD-DR variants were amplified by PCR from the plasmid DNA using the following composite primer pair: forward primer, 454 Titanium Lib-I primer A/5-base barcode/a2F; and reverse primer, 454 Titanium Lib-I primer B/a2R. Each sample was amplified in quadruplicate 20-μl PCR mixtures consisting of 10 ng of plasmid DNA, a concentration of 0.2 μM forward and reverse primers, and 1× Phusion high-fidelity (HF) master mix (New England BioLabs, Ipswich, MA). Thermal cycling consisted of an initial denaturation at 98°C for 30 s and 30 cycles of denaturation at 98°C for 10 s, annealing at 51.2°C for 30 s, and elongation at 72°C for 1 min, followed by a final extension at 72°C for 7 min. Following PCR, DNA amplicons were purified with the Agencourt AMPure XP PCR purification beads (Beckman Coulter, Indianapolis, IN), quantified using the Quant-iT PicoGreen double-stranded DNA (dsDNA) assay kit (Life Technologies, Grand Island, NY), and pooled in equimolar ratios at a final concentration of 30 ng/μl. Pyrosequencing was performed using the Roche 454 GS FLX Titanium chemistry (454 Life Sciences, Branford, CT) at the EnGenCore facility at the University of South Carolina, Columbia, SC.

### Analysis of ACCD-DR diversity.

The 454-generated sequence reads were analyzed with the QIIME software package (Quantitative Insights into Microbial Ecology), using the default parameters for each step (30). The sequences were chimera checked and clustered into ACCD-DR variant clusters using OTUpipe (27) at 99% sequence identity. Each ACCD-DR variant cluster (equivalent to an operational taxonomic unit [OTU] in 16S rRNA analysis) was represented by its most abundant sequence. A total of 33,625 quality-filtered reads were obtained for 51 samples, with an average of 659 reads per sample. The forward and reverse primers were removed using a customized script. Due to the many indels in the sequences, a custom script was employed to ensure the correct sequence length. Using the European Molecular Biology Open Software Suite (EMBOSS) water program (31), each 454 sequence trimmed of both primers was aligned to the P. cloacae ACCD-DR as the backbone sequence. If an insertion was found relative to the backbone, the insertion was deleted in the 454 sequence. If a deletion was found relative to the backbone, a gap was inserted into the 454 sequence at the corresponding position. The insertions in the 454 sequences were easy to identify; however, the contents of the gaps (i.e., the bases used to fill in the gaps) were impossible to determine within the limited context. Therefore, inevitably, a number of the resulting sequences still contained gaps. However, after this process, all sequences were of the same length, and the correct reading frame was maintained. The DNA sequences were translated into amino acid sequences, and amino acid sequences containing more than one unknown residue were excluded from the analysis. After quality filtering, 26,764 DNA sequences remained for 51 samples, with an average of 524 sequences per sample.

To calculate the between-sample (β) diversity between the ACCD-DR variant pools before and after each round of selection, we first standardized the sequence count per sample by rarefaction, so that each sample contained 80 sequences. A phylogenetic (neighbor-joining) tree was built from the representative sequences of the ACCD-DR variant clusters using Clustal W (32), and the tree was used to calculate β-diversity using the UniFrac distance metrics (33).

To calculate the frequency of each DNA base or amino acid residue at every DNA/protein position, variant counts were normalized across samples by frequency. The plyr (34) and reshape2 (35) packages in R version 2.15.0 (36) were applied to determine the DNA base/amino acid residue frequencies. The amino acid and DNA waffle plots were generated based on the frequencies of the residues and bases using the R package ggplot2 (37). The structure of the H26 ACCD-DR variant was computed using homology modeling on the SWISS-MODEL server (38) with the Pseudomonas sp. ACP ACCD (which bears the Q26 ACCD variant, PDB identification [ID] 1TYZ) as the template (23). The structures were visualized and aligned in PyMOL (www.pymol.org).

To identify the amino acid residues that were fixed or neutral in the selection assay, a linear regression was fitted in R to the frequencies of ACCD-DR variants in each library at time zero and after each round of selection for each residue at each position. A residue was defined as neutral if the linear regressions showed both positive and negative slopes in the three libraries and as fixed if it had a starting frequency of >0.1 and positive slopes in all three libraries.

To test whether eight fixed residues identified by our selection assay that lacked previously known function hitchhiked to fixation together, the chi-square test of independence and Fisher's exact test of independence were conducted in R to identify whether any two of the eight residues were associated with each other. In the ACCD-DR variant sequences at time zero before the competition assay, a binary code (0 for absence of the fixed residue and 1 otherwise) was used to indicate whether a base position contained the fixed residue or not. A log-linear model-based independence test was applied using the R package MASS to the 8-way contingency tables generated from the binary data for ACCD-DR variants in the three rhizosphere bacterial ACCD-DR libraries.

### Molecular evolution analyses of ACCD-DR pools.

We performed the codon-based Z-test (39) and Tajima's neutrality test (40) using the Molecular Evolutionary Genetics Analysis (MEGA 6.0) program (41) on the ACCD-DR DNA variants from all three rhizosphere bacterial ACCD-DR libraries at time zero. The ACCD-DR DNA variants were filtered by length and aligned using their protein-coding sequences in the EMBOSS water program, as described earlier. A total of 16,432 sequences were obtained for the molecular evolution analyses. The codon-based Z-test calculates the test statistic *dN-dS*, where *dS* and *dN* represent the synonymous and nonsynonymous substitutions per site, respectively. The data set was bootstrapped 500 times to estimate the variance, and the modified Nei-Gojobori method with Jukes-Cantor correction (assumed transition/transversion bias, 15) (42) was selected as the substitution model. Any position that contained alignment gaps or missing data was eliminated from pairwise sequence comparisons. The probability of rejecting the null hypothesis of strict neutrality (*dN* = *dS*) in favor of the alternative hypothesis (purifying selection with *dN* < *dS*) was measured and tabulated, and the level of significance was set at 5%. Tajima's neutrality test was conducted using all codon (1st, 2nd, and 3rd) positions, and all positions containing gaps and missing data were eliminated.

### Nucleotide sequence accession numbers.

The Illumina sequences are available in the European Nucleotide Archive under the study accession no. PRJEB11480. The 454 sequence reads from both the natural and artificial variant libraries are available in the European Nucleotide Archive under study accession no. PRJEB11657.

## RESULTS

### Diversity of ACC deaminase genes in the maize rhizosphere.

To assess whether maize rhizosphere soil was suitable as a source of ACCD protein variants, we first probed the genetic diversity of bacterial ACC deaminase genes. Rhizosphere bacterial ACC deaminase genes are GC rich and highly polymorphic (43), with a few widely conserved regions. Thus, we designed a degenerate primer pair to amplify a 113-bp region of the ACCD gene (Fig. 1). This region, the ACCD domain region (ACCD-DR), contains several conserved amino acid residues in the active site and some variable residues (21, 23, 24).

We assessed the variation in the ACCD-DR from our rhizosphere soil samples using paired-end Illumina sequencing. After quality filtering, clustering, and removal of singletons, we obtained >3.4 million different ACCD-DR DNA variants, which encode >450,000 different ACCD-DR protein variants. These numbers are likely inflated due to sequencing errors, but overall, the result indicates that the rhizosphere metagenome contains a high diversity of ACCD-DR variants. The seven most abundant ACC deaminase protein variants from this rhizosphere soil comprised 51.5% of the DNA sequences, and phylogenetic analysis indicated that they were likely encoded by members of the genera Burkholderia and Pseudomonas from the phylum Proteobacteria, and Tetrasphaera and Promicromonospora from the phylum Actinobacteria (see Fig. S1 in the supplemental material). Previous work has also shown members of the phyla Proteobacteria and Actinobacteria to express ACCD (44, 45). Importantly, the high level of ACCD protein diversity in rhizosphere soil indicated sufficient diversity to serve as an initial variant pool for a competition assay.

### Competition assay allows survival of functional ACCD-DR variants only.

To compete the ACCD-DRs expressed in E. coli, we required a competition assay in which the fittest ACCD-DR variants would be selected for and cheaters disallowed. In this assay, E. coli lacking a functional ACCD gene should not be able to survive by scavenging nitrogen released by adjacent strains that do have ACCD activity. To verify that this condition was met, we conducted a cheater test in which we compared E. coli cells with and without a functional ACCD. First, we were able to confirm the results of Li and Glick (28), who showed that E. coli transformed with a plasmid (p4U2) containing the ACCD gene from P. cloacae displayed ACCD activity and that it could grow with ACC as the sole nitrogen source. Second, we verified that E. coli cells containing the plasmid lacking the ACCD-DR (p4U2ΔACCD-DR) failed to grow with ACC as the sole nitrogen source (data not shown). Third, we mixed E. coli/p4U2 with E. coli/p4U2ΔACCD-DR in a 1:1 ratio and grew the mixed populations with ACC as the sole nitrogen source (see Fig. S2a in the supplemental material). After the first round of selection, both E. coli types were detected by PCR (see Fig. S2b in the supplemental material); however, E. coli/p4U2ΔACCD-DR was undetected after the second round of selection (see Fig. S2b in the supplemental material). This experiment confirmed that the competition assay would not allow the survival of cheater strains lacking ACCD.

### Initial diversity in the ACCD-DR variant libraries.

We amplified the bacterial ACCD-DR from a second rhizosphere DNA sample in three sets of five PCR replicates and cloned the pooled amplicons into p4U2 by domain swapping (Fig. 2). The three ACCD-DR libraries were transformed into E. coli grown in a minimal salt medium with ACC as the sole nitrogen source to select for successful transformants (Fig. 2). Libraries A, B, and C contained 891, 742, and 560 ACCD-DR DNA variant clusters (99% identity), which encoded 310, 268, and 226 ACCD-DR protein variants, respectively. In total, the libraries represented 1,220 unique DNA variants encoding 455 protein variants. Two explanations might account for a lower diversity of ACCD-DR variants in the rhizosphere libraries A, B, and C compared to that observed in the directly sequenced rhizosphere sample described above: (i) we generated the libraries in liquid culture, which reduced diversity due to competition among E. coli clones, and (ii) we sequenced the PCR amplicons of rhizosphere ACCD-DR variants directly without cloning them into E. coli, which allowed for the recovery of sequences that might have been lost due to PCR or cloning bias.

### Effect of selection on β-diversity of ACCD-DR pools.

Each library underwent six rounds of selection in triplicate. For each replicate, we collected samples prior to the competition assay (i.e., time zero) and samples after each of the 6 rounds of selection (Fig. 2).

**FIG 3:**
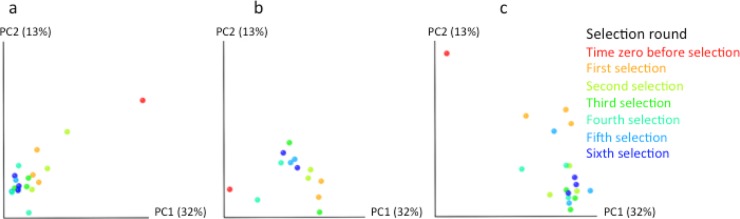
ACCD-DR variant pools cluster by selection round. The ACCD-DR variant pools after each round of selection for the three replicates within each library are clustered by round of selection in a principal coordinate analysis (PCoA) of the unweighted UniFrac distances between samples. The percent variation explained by the principal coordinates (PCs) is indicated on the axes. The ACCD-DR variant pools are colored by a gradient from red to blue, and each point corresponds to an ACCD-DR variant pool colored by selection round: red, time zero before selection; orange, cyan, green, aqua, teal, and blue, after the first to the sixth round of selection, respectively. Replicates for the same library are represented by dots of the same color for each round of selection. ACCD-DR variant pools are shown for library A (a), library B (note that this library contained two replicates only) (b), and library C (c). All panels are plotted on the same axes. Note that the major variation in the variant pools is between libraries; hence, the libraries cluster separately.

To gain a coarse overview of the impact of selection on the genetic diversity of the ACCD-DR gene pools, we estimated the β-diversity of the ACCD-DR pools from libraries A, B, and C using the unweighted UniFrac distance metric (33). The unweighted UniFrac metric ranges from 0 to 1, such that any two pools with closely related variants will have a low UniFrac value, while two pools with phylogenetically unique content will have a value closer to 1. Distances were computed for all pairwise comparisons between pools, and principal coordinate analysis (PCoA) of the UniFrac distance matrix was applied to display the relationships between pools. In all three libraries, the first round of selection strongly impacted diversity compared to the subsequent rounds of selection (Fig. 3). Only constructs in which the ACCD-DR is cloned in frame and forms a complete and functional ACCD gene are able to grow when ACC is the sole nitrogen source. Hence, nonfunctional constructs should be lost during the first round of selection, and this loss may explain the separation of the first round of selection from the subsequent selection rounds.

### Purifying selection fixed most essential residues in the rhizosphere metagenome.

To understand the specific effects of our competition assay on ACCD-DR diversity, we analyzed previously reported essential amino acid residues (21, 23, 24). Most essential residues (G20, Q23, S24, N25, T27, R28, A34, and A35) that are involved in binding cofactor and substrate and ACCD monomer-monomer interaction (21) were already fixed in the starting libraries at >90% frequency, suggesting that strong selection pressures existed in the rhizosphere bacterial populations. To confirm this supposition, we used the codon-based Z-test (39) and calculated Tajima's D value (40) (see Table S1 in the supplemental material). A negative Tajima's D value of −2.380024 indicated the presence of purifying selection in the starting library, and the subsequent codon-based Z-test results (*P* < 0.05) supported rejection of the null hypothesis of strict neutrality in favor of the alternative hypothesis of purifying selection. Hence, nature had already selected the function of the ACCD-DR variants in rhizosphere bacteria, thereby providing a starting pool to optimize and understand the functionality of the less-constrained residues in the ACCD-DR.

### Selection on a divergent essential residue.

**FIG 4:**
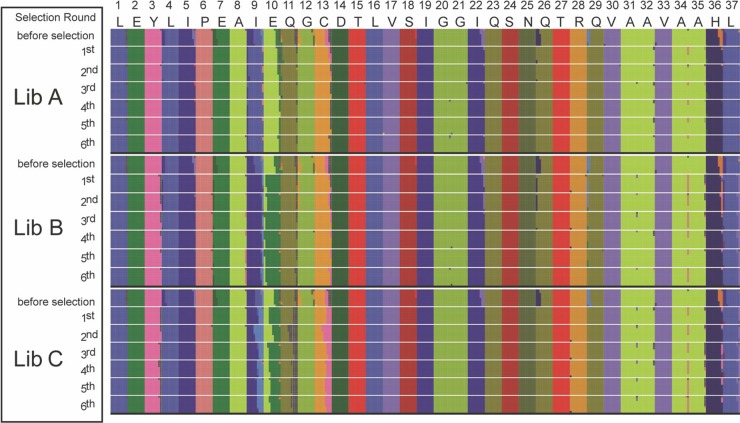
Amino acid residue waffle plots for libraries (Lib) A, B, and C. The amino acid residue waffle plots show the frequency of each residue in ACCD-DR variant pools at time zero before selection and after each round of selection averaged for the three replicates in each library. Each amino acid residue is represented by a unique color, and the percentage of grids of the same color shows the frequency of that residue at a position. The amino acid residues on top of the waffle plots are color coded and show the sequence in the P. cloacae ACCD-DR. The number above each amino acid residue shows the position of the residue from 1 to 37. All amino acid residues are uniquely colored to show their relative abundances.

One essential site previously identified by structural analyses was not fixed in our starting library. In a small proportion of the starting library, residue 26 contained a divergent amino acid that is present in some rhizosphere bacterial ACCD sequences (Fig. 1). Karthikeyan et al. (23) reported that Q26 interacts with the bound sulfate in the active site of the protein and is important for ACCD function. In all three libraries, both glutamine (Q) and histidine (H) were present at residue 26 in the population prior to selection, but glutamine was fixed or enriched after the first round of selection (Fig. 4).

**FIG 5:**
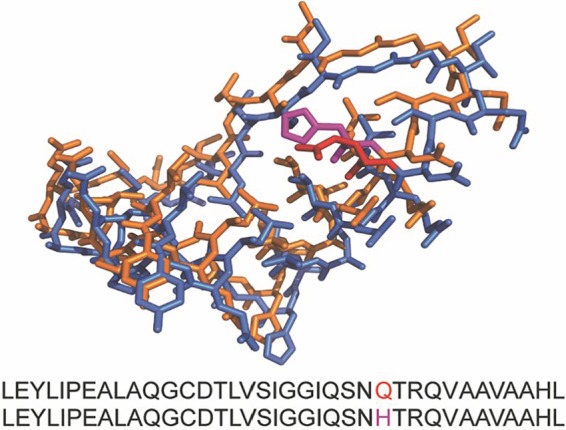
Alignment of the three-dimensional (3D) structures for Pseudomonas sp. ACP ACC deaminase and homology-modeled ACCD-DR. The alignment of the Pseudomonas sp. ACP ACCD-DR is shown in blue, and the homology-modeled ACCD-DR based on the Pseudomonas sp. ACP ACC deaminase structure is shown in orange. The Q26 residue in the Pseudomonas sp. ACP ACCD-DR is colored in red, and the H26 residue in the homology-modeled ACCD-DR is colored in purple. The other regions of the full-length ACC deaminase protein structures from these two variants are identical and were omitted in the alignment.

To assess why the H26 ACCD-DR variant was excluded quickly from the variant pool by the selection assay, we employed homology modeling to estimate the structure of this variant. We used the known Pseudomonas sp. ACP ACCD crystal structure (PDB ID 1TYZ), which encodes the Q26 ACCD-DR variant, as the template to model the structure of the H26 variant. We found that these two structures were very similar when aligned (Fig. 5). However, because the side chain of the glutamine residue interacts with the bound sulfate ion in the active site of the protein (21, 23, 24), a change from an uncharged to a charged amino acid may impact the efficiency of the deaminase in the competition assay. Thus, it appears that while Q is more beneficial for binding ACC in E. coli, H may favor an alternative substrate or context in the rhizosphere.

### Competition assay reveals important nonessential residues.

Our competition assay acted on nonessential sites, suggesting that they are important for function. ACCD-DR variants with a leucine (L) at residue 4 were enriched after one or two rounds of selection in all libraries (Fig. 4). Yao et al. (21) reported that this residue is located on α-helix 3 in the Hansenula saturnus ACCD, is in close contact with α-helix 2, and binds the PLP cofactor. Although the underlying structural mechanism is not clear, this functionality may explain why the L4 ACCD-DR variants were enriched by our competition assay.

We observed that other sites with completely unknown roles in ACCD function were fixed in the competition assay. Residues I5, E7, G12, C13, I22, Q29, H36, and L37 were fixed at the first round of selection. Based on the known structure of the yeast ACCD protein, which is highly similar to the bacterial ACCD structure, residue I22 is on a loop between β-strand C and α-helix 4 of the protein and is involved in linking the active-site cavity to the surface of the protein (21). The ACCD consists of two domains (23), a small domain of unknown function and the cofactor-binding domain. As components of the small domain, residues I5, E7, G12, C13, Q29, H36, and L37 may help maintain the overall shape of the protein during the enzymatic degradation of ACC (21, 24).

Given the proximity of many of these residues to each other, we tested the time zero (“before selection”) ACCD-DR variants for independence among the eight residues to determine if selection at one residue was accompanied by concomitant changes at another site. Our results indicated that these residues were significantly associated (*P* < 10e−16). To further elucidate which subsets of the eight residues were likely to be selected together in our assay, we employed statistical coupling analysis (SCA) (46) of the time zero rhizosphere bacterial ACCD-DR variants (see Fig. S3 in the supplemental material). SCA calculates the sequence similarities of ACCD-DR variants based on the multiple-sequence alignment of the time zero ACCD-DR variants and constructs a positional correlation matrix of all residues in the ACCD-DR. All residue pairs within the fixed residues (residues 4, 5, 7, 12, 13, 22, 26, 29, 36, and 37) were more correlated than others. Thus, we could not exclude the possibility that these residues went to fixation together. As these residues are scattered throughout α-helices 4 and 5 and the loops connecting α4, α5, and β-sheet 3, it may be that these residues cooperatively increase the function of the ACCD.

While most ACCD studies have focused on the PLP-binding domain of ACCD, our results reveal that residues in other parts of the protein, especially the small domain, are also critical for the optimal efficiency of the enzyme. Therefore, our competition assay was able to reveal additional sites, or the collective importance of these sites, impacting the functional performance of ACCD in E. coli.

### Neutral sites unaffected by the competition assay.

Other sites remained heterogeneous throughout the selection process. Residues 9 and 10, for example, bore a mixture of several residues (predominantly IE and LA) prior to selection in library A. After the first round of selection, ACCD-DR variants with the LA residues began to dominate the population, although IE variants were still present in the population at a much lower frequency (Fig. 4). Similarly, the IE ACCD-DR variants in library B became dominant after the first round of selection (Fig. 4). The ACCD-DR variants from library C contained a mixture of I/L/M and A/E at residues 9 and 10, respectively, prior to selection, and there was no clear winner after six rounds of selection (Fig. 4). Together, these data suggest that LA and IE do not differentially affect the function of the ACCD-DR.

To test this hypothesis, we constructed the LA and IE ACCD-DR protein variants on identical backgrounds, so that the variants only differed at the 9th and 10th residues, and grew E. coli cells containing either variant separately on ACC as the sole nitrogen source. We did not observe any significant differences in their growth rates (see Fig. S4 in the supplemental material), supporting the neutrality of these two residues with respect to the efficiency of ACCD in E. coli.

Neither residue 9 nor 10 is known to be involved in the enzymatic actions of ACCD. Modeling of the two “winning” ACCD-DR variants at positions 9 and 10 showed that the structure of the IE ACCD-DR variant was very similar to that of the LA variant (data not shown). Furthermore, the predicted structures indicated that the IE and LA residues are located on the outside of the protein structure, away from the active site. Thus, their location may explain their neutral behavior under the selection conditions.

Similarly, we found that residue 11 remained heterogeneous throughout the selection assay. Based on its position in the ACCD-DR, we predict that this residue has no direct role in ACCD function. Hence, the heterogeneity maintained at sites 9, 10, and 11 may reflect the neutrality of these residues in the competition assay and in the function of ACCD.

### Selection at the DNA level.

We analyzed the selection of ACCD-DR variants at the DNA level (see Fig. S5 in the supplemental material). As expected, we found that the most variation was in the wobble positions of the codons, and variation in the first and second positions of the codons was fixed quickly after the first round of selection. Reflecting the observations at the protein level, amino acid residues that were highly varied throughout the selection assay displayed persistent polymorphisms in the first and second codon positions after several rounds of selection.

Most essential and important residues (e.g., see Fig. S5 in the supplemental material, G12 and S24 in the purple rectangles) contained more than one DNA variant for each residue at time zero, and multiple codons encoding the same amino acid residue were fixed in the competition assay in the three libraries, indicating that selection from nature and our assay acted mainly at the protein level. However, two essential residues, G20 and N25, contained only one dominant DNA variant (GGC and AAC, respectively) in all three libraries at time zero, revealing selection from nature on synonymous codons. Importantly, these codons are the preferred codons for Pseudomonas, Burkholderia, and Promicromonospora (47–49). We also observed enrichment of the codons for the neutral residues 9, 10, and 11 (see Fig. S5 in the supplemental material, yellow rectangle) in our competition assay. With the exception of 11Q, the enriched codon in each case is the dominant codon in the Pseudomonas, Burkholderia, and Promicromonospora genomes (47–49).

We also observed some rare codons for E. coli in the ACCD-DR libraries, the most prominent being the codon CCC encoding proline at residue 6, along with other examples, such as the codon CTC encoding leucine at residue 1, the codon TTG encoding leucine at residue 16, and the codon TTG encoding leucine at residue 37 (49). These observations may reflect the rhizosphere bacterial origin of ACCD-DR variants: for example, codons CCC and CTC are common codons in Pseudomonas, Burkholderia, and Promicromonospora (47–49).

### Comparison with an artificial ACCD-DR variant pool.

To validate the findings from the rhizosphere-derived libraries, we sought to conduct the selection assay on the nonnatural ACCD-DR variant library. To this end, we constructed an artificial ACCD-DR variant library generated from doped DNA oligomer synthesis by using one of the winning LA ACCD-DR DNA variants as the wild-type backbone, and we doped each base with 2.1% non-wild-type nucleotides. Compared to the rhizosphere bacterial ACCD-DR variant libraries that started with 1,262 unique DNA variants encoding 471 protein variants in total, our artificial ACCD-DR variant pool started with 932 unique ACCD-DR DNA variant clusters at 99% similarity, which encoded 684 unique ACCD-DR protein variants. Thus, the artificial library was composed of a similar number of variants as our rhizosphere bacterial ACCD-DR variant library.

We selected the artificial ACCD-DR variant library for six rounds and sequenced the ACCD-DR variants at time zero before the selection and after each round of selection, as performed for the rhizosphere microbiome-based libraries. Using the amino acid waffle plots to track the selection at the amino acid level and the same cutoff values to identify important residues enriched by the competition assay, we found that the L4 residue was important and was fixed after the first round of selection (see Fig. S6 in the supplemental material). Similarly, although the H residue competed with Q at the 26th position, the other fixed and neutral residues observed in the rhizosphere bacterial ACCD-DR variant libraries were already the dominant residues in the artificial ACCD-DR variant libraries at time zero before the selection assay. Thus, the artificial protein variant pools yielded results similar to those of the natural pool.

## DISCUSSION

**FIG 6:**
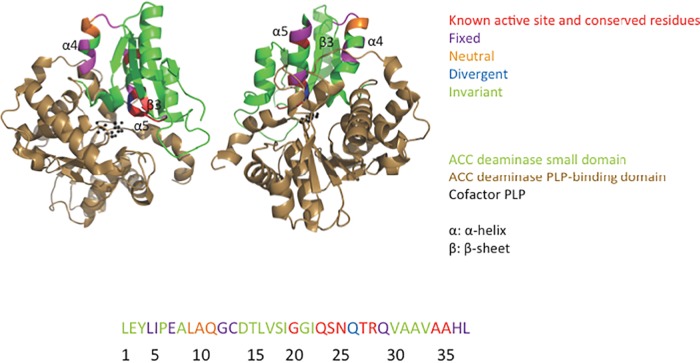
Summary of the selection assay results. The Pseudomonas sp. ACP ACC deaminase monomer as shown in Fig. 5 is shown in two different orientations. The small domain is displayed in green, and the PLP-binding domain is colored in copper. The cofactor PLP is shown in black, and the α-helices and β-sheet harboring the ACCD-DR are marked. The essential, fixed, neutral, and divergent residues identified by the selection assay within the ACCD-DR are colored red, purple, orange, and blue, respectively, and the invariant residues are shown in green. The linear amino acid sequence of ACCD-DR with the above-mentioned five types of residues is colored accordingly below the protein structure.

Protein structure analysis and optimization have traditionally been arduous and low-throughput processes requiring the generation of purified proteins and point mutation libraries. Even deep mutational scanning, which provides a way to simultaneously assay hundreds of thousands of variants, cannot exhaustively explore higher-order mutational space. For some applications, the sequence space of natural proteins is more applicable and relevant; therefore, we tested whether alleles naturally present in rhizosphere microbial DNA could be used in a selection assay. We employed a rhizosphere microbiome-derived gene amplicon library to complement the active domain region of the ACCD gene and competed the variants in a growth-based selection assay. Sequence analysis of the surviving variants allowed us to study the sequence-function relationships of ACCD-DR, make evolutionary inferences of selective pressures on the protein, and identify optimal ACCD-DR variants in our E. coli-based growth assay (Fig. 6). Together, these initial observations underscore that environmental metagenomes present an avenue for protein optimization when genetic engineering approaches are not desirable.

Although our study captured only a portion of the functional ACCD-DR variants that exist in the soil rhizosphere, our assay was able to identify functional, divergent, and neutral residues of the ACCD-DR. Many of the previously identified essential residues within the ACCD-DR were fixed in our starting libraries before the competition assay, and we observed evidence of purifying selection on these sites. Other sites not previously reported to be essential showed a preference for particular amino acids in our competition assay. Moreover, our assay revealed combinations of amino acid residues that appear to be selected in concert and may be critical to the optimal efficiency of ACCD. Of note, these residues are within the previously structurally identified but less-studied small domain of ACCD. Most surprisingly, the assay uncovered diversification at Q26, an essential residue within the ACCD-DR. While the selection assay favored one of two dominant residues, Q over H, at position 26, the presence of this alternative residue in nature suggests that other selective pressures, such as the need for flexibility for alternative substrates or the need to coevolve with other residues in the protein, may be driving diversification at this residue.

Finally, we discovered codon bias within the essential residues G20 and N25 and the neutral residues 9I, 10E, and 11Q. Interestingly, except for 11Q, the dominant codon in each case is the most prevalent codon across the genome in those species most likely contributing the ACCD genes from the rhizosphere, Pseudomonas, Burkholderia, and Promicromonospora (codon usage has not yet been calculated for Tetrasphaera) (47–49). Why the CAA codon for 11Q is fixed is unknown. In Pseudomonas, Burkholderia, Promicromonospora, and E. coli genomes, the CAG codon is preferred for glutamine. The use of nonoptimal codons is believed to contribute to translation kinetics and cotranslational folding (50–52). To this point, 11Q is the first amino acid in the linker region between α4 and β3. In contrast, the other Gln residues, 23Q, 26Q, and 29Q, in the ACCD-DR all use the major CAG codon.

Our ACCD-DR artificial protein variant pools yielded similar results, supporting the use of a microbiome variant pool for mutational analysis and protein optimization. While a larger synthetic ACCD-DR variant library may reveal deeper insights into the sequence-function relationships within the ACCD-DR, a microbiome-derived library may more applicable in agricultural engineering. In addition, microbiome amplicon libraries have a key advantage over synthetic libraries in that they avoid the large nonfunctional space of every possible protein variant: microbiome allele amplicon libraries are enriched for combinations of functional polymorphisms, which are sparse in randomly mutagenized libraries. Furthermore, for agricultural needs, discovering a protein variant already present in the environment may speed lab-to-field use and avoid genetic engineering approaches, if needed. Alternatively, it may be possible to trace the protein variant back to its host bacterium and biostimulate this bacterium in the environment without the need for crop genetic manipulation (17) or bioaugmentation (53).

Overall, this work shows that the generation of protein variant pools from the rhizosphere soil metagenome can provide a sketch of the functional regions of a protein domain; this is a starting point for understanding protein structure and optimizing enzyme performance. Compared to the generation of protein variant pools from artificial libraries, this alternative approach focuses on the natural and functional protein variant sequence space while still assessing thousands of variants in a high-throughput format. The growth-based selection assay is straightforward and readily adaptable to other enzymes and expression hosts. Hence, the use of microbiome-derived amplicon libraries in a competition assay has the potential to speed the translation of novel natural products from nature to industry.

## Supplementary Material

Supplemental material
