# Supplementary material for: Novel Rhizosphere Soil Alleles for the Enzyme 1-Aminocyclopropane-1-Carboxylate Deaminase Queried for Function with an In Vivo Competition Assay
Source: Appl Environ Microbiol. 2016 Feb 5;82(4):1050–9. doi: 10.1128/AEM.03074-15 (PMC4751833; doi:10.1128/AEM.03074-15)
Supplement: Supplemental material [file supp_82_4_1050__index.html]

Novel Rhizosphere Soil Alleles for the Enzyme 1-Aminocyclopropane-1-Carboxylate Deaminase Queried for Function with an In Vivo Competition Assay — Supplemental material 

# Novel Rhizosphere Soil Alleles for the Enzyme 1-Aminocyclopropane-1-Carboxylate Deaminase Queried for Function with an *In Vivo* Competition Assay

## Supplemental material

- Supplemental file 1 -

  Accession numbers for sequences listed in Fig. 1, results from Tajima's neutrality test (Table S1), seven most abundant rhizosphere bacterial ACCD-DR protein variants (Fig. S1), inability of nonfunctional ACCD-DR variants to grow on ammonia produced by other ACCD-DR variants (Fig. S2), positional correlation of time zero rhizosphere bacterial ACCD-DR variants (Fig. S3), growth curves (Fig. S4), DNA base waffle plots (Fig. S5), and amino acid residue waffle plots for the artificial ACCD-DR protein variant pools at time zero and after each round of selection (Fig. S6).

  PDF, 1.6M
