# Supplementary material for: Novel Rhizosphere Soil Alleles for the Enzyme 1-Aminocyclopropane-1-Carboxylate Deaminase Queried for Function with an In Vivo Competition Assay
Source: Appl Environ Microbiol. 2016 Feb 5;82(4):1050–9. doi: 10.1128/AEM.03074-15 (PMC4751833; doi:10.1128/AEM.03074-15)
Supplement: Supplemental material [file AEM.03074-15_zam999116907so1.pdf]

Sequence identifiers for sequences listed in Figure 1:
Fusarium graminearum (XP_011323515.1), *Neosartorya fischeri* (XP_001265664.1), *Aspergillus flavus* (KJJ31062.1), *Penicillium citrinum* (BAA92150.1), *Hansenula saturnus* (Q7M523.1), *Pseudomonas fluorescens* (ABE66284.1), Uncultured bacterium (ADN88184.1), *Enterobacter cloacae* (AAD05069.1), *Pseudomonas putida* (ABJ91236.1), *Klebsiella oxytoca* (ACJ12921.1), *Variovovrax paradoxus* (WP_042577431.1), *Burkholderia terricola* (ACH81529.1), *Agrobacterium tumefaciens* (AAK28496.1), *Methylobacterium sp.* (ACH58597.1), *Phyllobacterium brassicacearum* (ABO31418.1), *Methylobacterium nodulans* (WP_015931930.1), *Arabidopsis thaliana* (AAF79717.1), *Microbulbifer degradans* (ABD80923.1).

<table>
<tr><th>Sequences (m)</th><th>Number of segregating sites (S)</th><th>S/m (ps)</th><th>ps/a1 (Θ)</th><th>Nucleotide diversity (Π)</th><th>Tajima’s D</th></tr>
<tr><td>16433</td><td>110</td><td>0.964912</td><td>0.093824</td><td>0.004721</td><td>-2.38002</td></tr>
<tr><td colspan="6">NOTE.-- The analysis involved 16433 nucleotide sequences. Codon positions included were 1st+2nd+3rd. All ambiguous positions were removed for each sequence pair. There were a total of 114 positions in the final dataset. Evolutionary analyses were conducted in MEGA6.</td></tr>
</table>

Supplementary Table 1. Results from Tajima's neutrality test on the rhizosphere bacterial ACCD-DR variants from Libraries A, B, and C at time zero before selection.

L	E	Y	L	I	P	E	A	I	E	Q	G	C	D	T	L	V	S	I	G	G	I	Q	S	N	Q	T	R	Q	V	A	A	V	A	A	H	L	
								L	A																												*Burkholderia kururiensis*
				V		D			A	K		A									Y				H										R		*Tetrasphaera elongate*
									A	S																											*Pseudomonas sp. GM60*
								L	A	E											Y																*Burkholderia fungorum*
				V				L	A			A									Y				H												*Promicromonospora sukumoe*
									A	A																											*Pseudomonas sp. GM60*
						D		L																													*Burkholderia caryophylli*

Supplementary Figure 1. The seven most abundant rhizosphere bacterial ACCD-DR protein variants. Rhizosphere bacterial ACCD-DRs were amplified by PCR from the pooled DNA sample of four Missouri maize rhizosphere soil samples and sequenced with Illumina paired-end sequencing. The first amino acid sequence shows the ACCD-DR from the *P. cloacae* ACCD. Below this sequence, the seven most abundant ACCD protein variants found in this DNA sample are shown. Listed at right are the top protein BLAST hits for these protein variants. Sequence identifiers of these hits: *Burkholderia kururiensis* (EU886318.1), *Tetrasphaera elongate* (WP_010850705.1), *Pseudomonas sp. GM60* (EJM76881.1), *Burkholderia fungorum* (ACH81538.1), *Promicromonospora sukumoe* (WP_020018078.1), *Burkholderia caryophylli* (ACH81522.1).

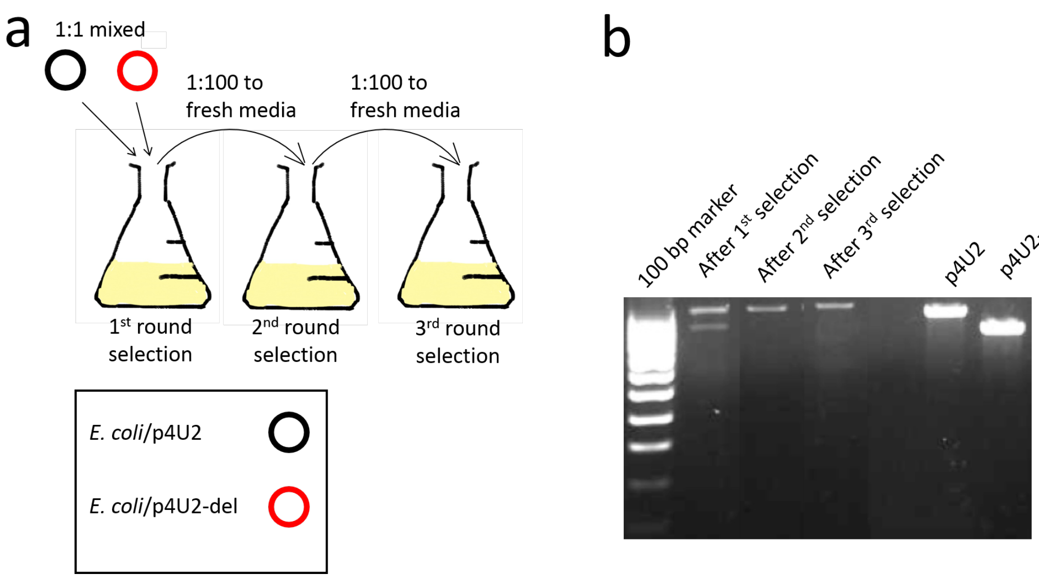

Supplementary Figure 2. Non-functional ACCD-DR variants cannot grow on ammonia produced by other ACCD-DR variants. (a) *E. coli* containing the *P. cloacae* ACC deaminase on the plasmid (p4U2, black circle) were mixed in a 1:1 ratio with *E. coli* containing the plasmid lacking the ACCD-DR (p4U2-del, red circle). The two variants were grown in the DF/ACC media for three rounds of selection. (b) The ACCD-DR was amplified by PCR from the mixed growth samples. PCR amplicons from p4U2 and p4U2-del were used as controls.

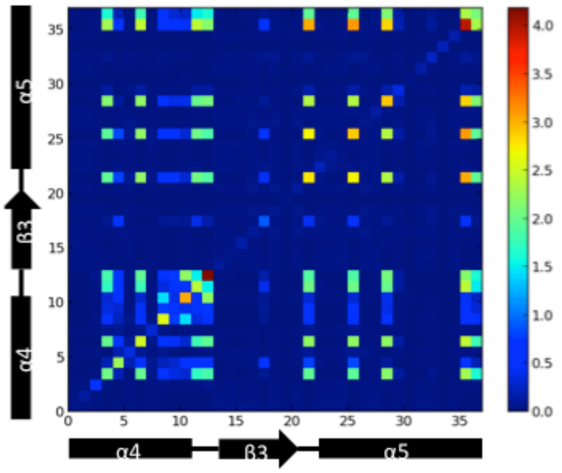

Supplementary Figure 3. Positional correlation of time zero rhizosphere bacterial ACCD-DR variants. Warmer colors in the heatmap indicate a stronger correlation between residue pairs. The α-helices and β-sheet of ACCD-DR are listed beside and under the heatmap.

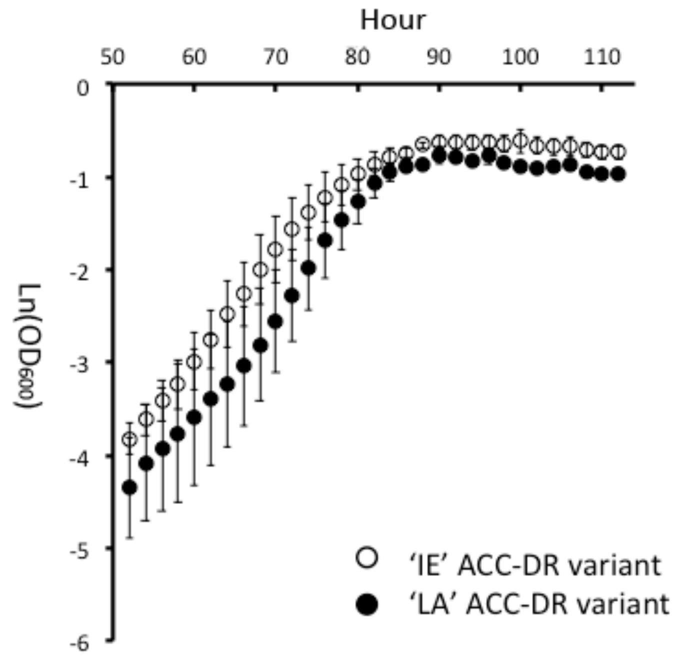

Supplementary Figure 4. Individual growth curves of *E. coli* cells containing 'IE' and 'LA' ACCD-DR variants in ACC/DF medium. Open circle: *E. coli* containing the 'IE' ACC deaminase protein variant. Closed circle: *E. coli* containing the 'LA' ACC deaminase protein variant. These two ACCD-DR protein variants were identical in other residues except for the 'IE' or 'LA' residues at positions 9 and 10. The curves were generated by plotting the natural log of the OD_{600} values of the variants versus time. The error bars represent the standard errors of the mean from five replicates for each strain.

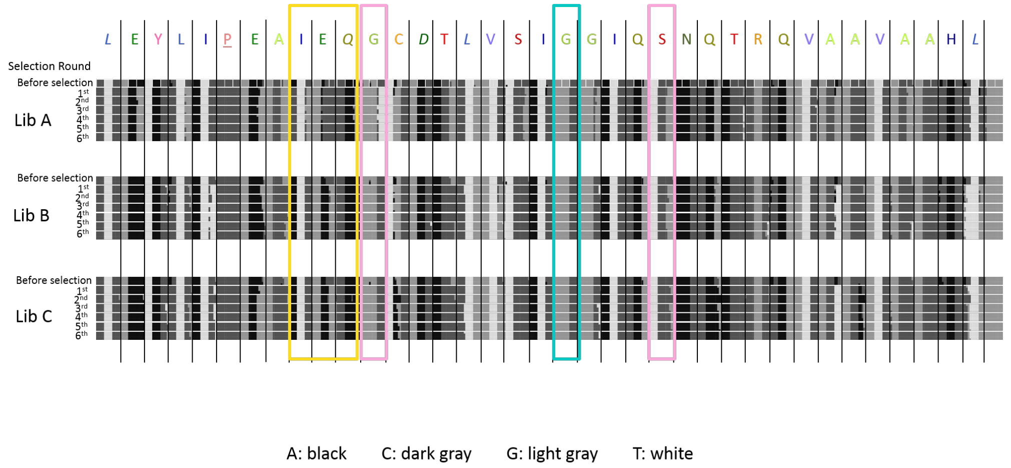

Supplementary Figure 5. DNA base waffle plots for Libraries A, B, and C ACCD-DR DNA variant pools at time zero and after each round of selection. The DNA base waffle plots for each Library were made based on the frequency of each base in ACCD-DR DNA variant pools at time zero and after each round of selection averaged for the three replicates in each library. Each base is represented by a unique color, and the percentage of grids of the same color shows the frequency of that base at a position. Bases A, C, G, and T are represented in black, dark gray, light gray, and white, respectively. The amino acid sequence from the *P. cloacae* ACCD-DR is shown on top of the waffle plots, and P6 is underlined to indicate a possible rare codon. DNA encoding amino acid residues 9-11, 12, 20 and 24 are shown in the cyan, yellow, and purple rectangles to show examples of fixed, neutral, and essential residues, respectively.

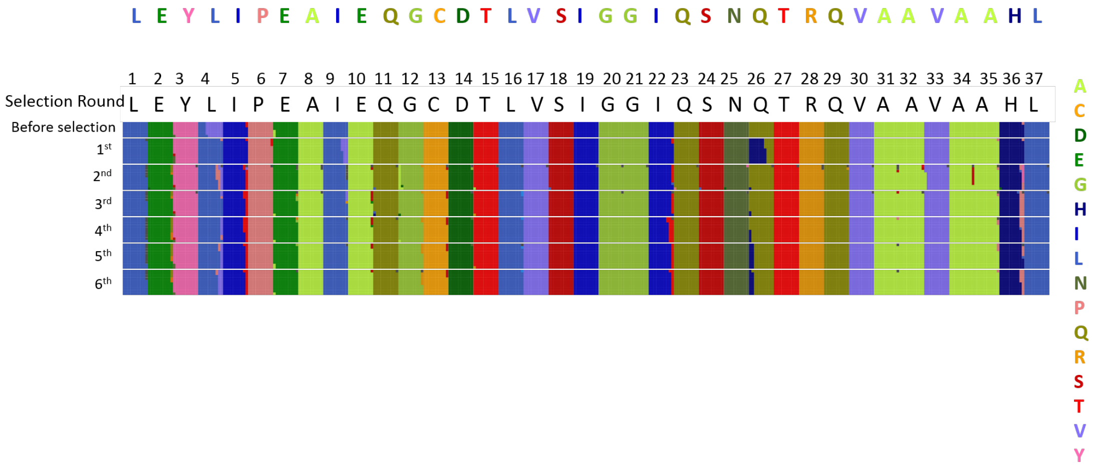

Supplementary Figure 6. Amino acid residue waffle plots for the artificial ACCD-DR protein variant pools at time zero and after each round of selection. The amino acid residue waffle plot is shown as in Figure 4.
